# Cost-Effectiveness Analysis of Pharmacogenomics-Guided Versus Standard Dosing of Warfarin in Patients with Mechanical Prosthetic Heart Valve

**DOI:** 10.5812/ijpr-143898

**Published:** 2024-05-14

**Authors:** Homa Hemati, Marzieh Nosrati, Mandana Hasanzad, Parham Rahmani, Soroush Fariman, Mohadese Sarabi, Sepideh Shirvani, Parham Sadeghipour, Shekoufeh Nikfar

**Affiliations:** 1 Department of Pharmacoeconomics and Pharmaceutical Administration, Faculty of Pharmacy, Tehran University of Medical Sciences, Tehran, Iran; 2 Personalized Medicine Research Center, Endocrinology and Metabolism Clinical Sciences Institute, Tehran University of Medical Sciences, Tehran, Iran; 3 Medical Genomics Research Center, Tehran Medical Sciences Islamic Azad University, Tehran, Iran; 4 Division of Pharmaceutical Outcomes and Policy, Eshelman School of Pharmacy, University of North Carolina Chapel Hill, NC, USA; 5 Cardiovascular Intervention Research Center, Rajaie Cardiovascular Medical and Research Center, Iran University of Medical Sciences, Tehran, Iran

**Keywords:** Pharmacogenomics, Precision Medicine, Quality-Adjusted Life Year, Heart Valve, Pharmacoeconomics, Cardiovascular Disease, Economic Evaluation, Warfarin, Cost Effectiveness

## Abstract

**Background:**

Warfarin is the only approved anticoagulant for antithrombotic treatment in patients with mechanical prosthetic heart valves (MPHV). However, dosing warfarin is challenging due to its narrow therapeutic window and highly variable clinical outcomes. Both low and high doses of warfarin can lead to thrombotic and bleeding events, respectively, with these complications being more severe in individuals with sensitive genetic polymorphisms. Incorporating genetic testing could enhance the accuracy of warfarin dosing and minimize its adverse events.

**Objectives:**

This study aims to evaluate the utilities and cost-effectiveness of pharmacogenomics-guided versus standard dosing of warfarin in patients with MPHV in Iran.

**Methods:**

In this economic evaluation study, a cost-effectiveness analysis was conducted to compare pharmacogenomics-guided versus standard warfarin dosing. Data related to quality of life (QoL) were collected through a cross-sectional study involving 105 randomly selected MPHV patients using the EuroQol-5D (EQ-5D) Questionnaire. Costs were calculated with input from clinical experts and a review of relevant guidelines. Additional clinical data were extracted from published literature. The pharmacoeconomic threshold set for medical interventions within Iran's healthcare system was $1,500. A decision tree model was designed from the perspective of Iran's healthcare system with a one-year study horizon. Sensitivity analyses were also performed to assess the uncertainty of input parameters.

**Results:**

The utility scores derived from the questionnaire for standard and pharmacogenomics-guided warfarin treatments were 0.68 and 0.76, respectively. Genotype-guided dosing of warfarin was more costly compared to the standard dosing ($246 vs $69), and the calculated incremental cost-effectiveness ratio (ICER) was $2474 per quality-adjusted life year (QALY) gained. One-way sensitivity analyses showed that our model is sensitive to the percentage of time in the therapeutic range (PTTR), the cost of genetic tests, and the utility of both pharmacogenomics-guided and standard dosing arms. However, the probabilistic sensitivity analysis demonstrates the robustness of our model.

**Conclusions:**

Warfarin dosing with pharmacogenomics testing is currently not cost-effective. However, if the cost of genotyping tests decreases to $118, the ICER would become cost-effective.

## 1. Background

Valvular heart disease (VHD) is a significant contributor to cardiovascular morbidity and mortality worldwide and is projected to increase in the coming decades, with rheumatic heart disease (RHD) being the leading cause of VHD in developing countries (1). The incidence of RHD remains high, with 60% of patients with RHD developing VHD annually (2). Heart valve replacement surgery, the second most common cardiac surgery, is crucial in treating patients with VHD and reducing the burden of disease and mortality (3). There are two types of prosthetic valves: Mechanical and bioprosthetic. Regardless of the valve type, patients undergoing heart valve replacement require antithrombotic therapy to reduce the risk of thromboembolic complications (4-6).

Patients with mechanical prosthetic heart valves (MPHV) need lifelong anticoagulant therapy, with warfarin being the only approved anticoagulant agent (4). Warfarin, an oral anticoagulant, inhibits the vitamin K epoxide reductase complex 1 (VKORC1) and prevents the activation of vitamin K, playing a critical role in the prevention of thromboembolism (3). Despite its importance, managing warfarin therapy is challenging due to its narrow therapeutic window and the considerable variability in dose response among patients (7). Out-of-range International Normalized Ratio (INR) levels are associated with an increased risk of thromboembolic and bleeding events. Consequently, patients must undergo regular monitoring to ensure their INR levels remain within the therapeutic range.

Variations in warfarin metabolism due to genetic differences have significant clinical implications. Polymorphisms in genes such as cytochrome P450 CYP2C9 and vitamin K epoxide reductase VKORC1 (3) can significantly affect the required dose of warfarin and increase the risk of adverse events, particularly bleeding. Patients with specific polymorphisms in these genes tend to experience greater fluctuations in their INR levels (7), and are at a higher risk of bleeding during the initial phase of anticoagulant therapy (8-14). Patients carrying CYP2C9^2^ and CYP2C9^3^ polymorphisms metabolize S-warfarin more slowly and require a lower warfarin dose (10, 15). Similarly, patients with one or two VKORC1 A haplotypes require lower warfarin doses (16). Many studies showed the association between the mentioned genes' polymorphisms and interpatient dose-response variation of warfarin. In 2007, the US Food and drug administration suggested pharmacogenomics testing (PGx) and added dose recommendations considering CYP2C9 and VKORC1 genotypes to identify better a therapeutic warfarin dose for each patient (4, 17). The Clinical Pharmacogenetics Implementation Consortium (CPIC) has further supported this approach by developing guidelines for PGx-guided dosing of warfarin, emphasizing the integration of genetic information in determining the optimal warfarin dose for individual patients (7).

However, the economic implications of PGx-guided dosing versus standard dosing remain a subject of debate. The initial costs of genetic testing pose an additional financial burden on healthcare systems, particularly in developing countries like Iran where healthcare budgets are constrained.

## 2. Objectives

This economic evaluation aims to assess whether the benefits of pharmacogenomics-guided dosing, in terms of improved patient outcomes and potentially reduced adverse events, justify the additional costs. By providing a thorough cost-effectiveness analysis, this study seeks to offer valuable insights that could influence healthcare policy and contribute to more effective resource allocation in the management of patients with MPHV on warfarin therapy.

## 3. Methods

### 3.1. Decision Model

**Figure 1.**
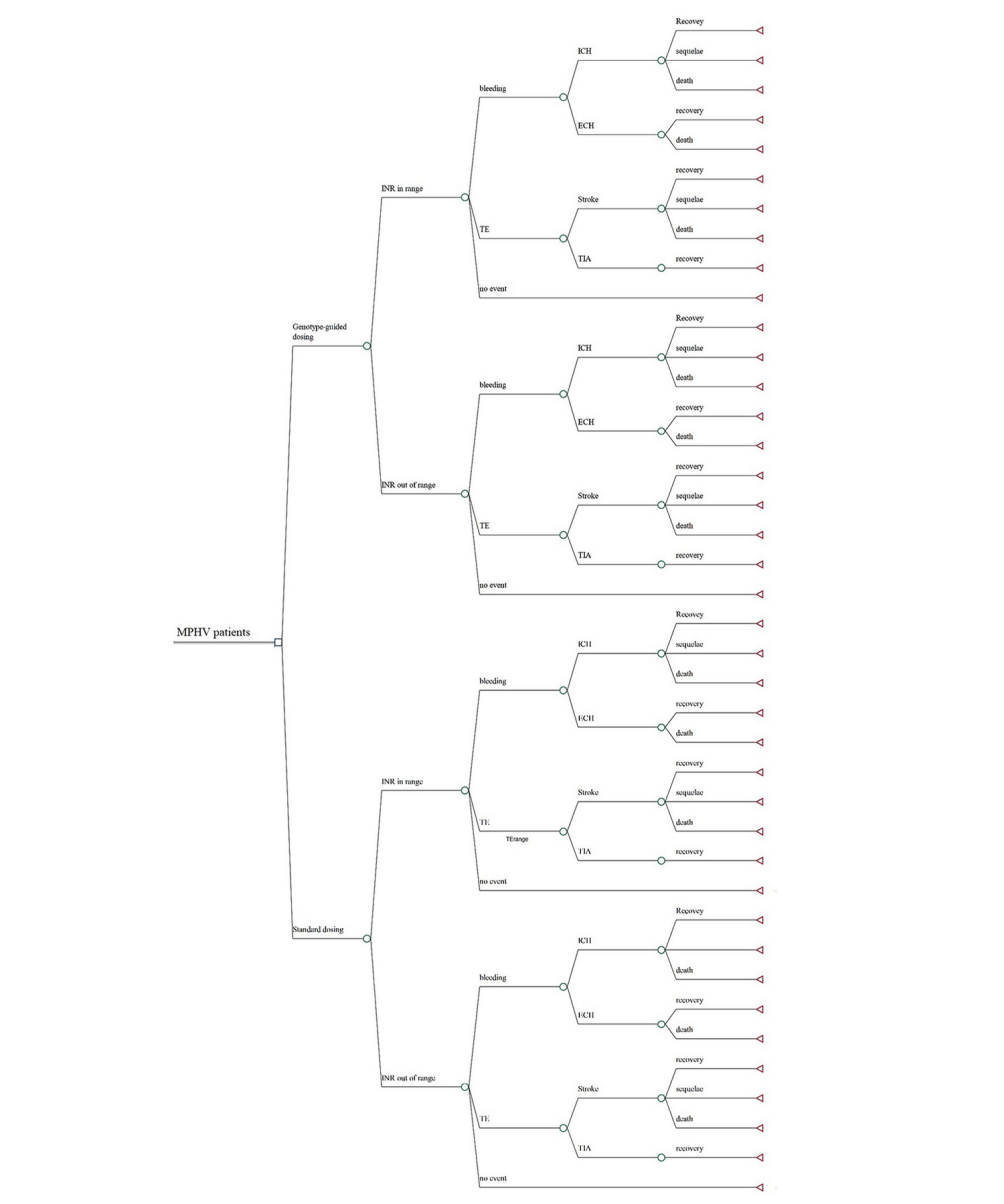
Decision tree model of dosing strategies for patients with MHV initiating warfarin therapy. ECH, extracranial haemorrhage; ICH, intracranial haemorrhage; INR, international normalized ratio; MPHV, mechanical prosthetic heart valve replacement; TE, thromboembolism; TIA, transient ischemic attack.

In this study, we conducted a cost-utility analysis to compare the outcomes and costs of two treatment strategies: Standard versus genotype-guided warfarin management over a 1-year time horizon (Figure 1). Given the limited time frame, we developed a decision tree model using MS Excel 2022. For the base-case analysis, a hypothetical cohort of 1000 Iranian patients aged 18 or above, who have recently undergone MPHV surgery and have no contraindications to warfarin administration, was used.

In the standard management group, the warfarin dose is calculated using a standard algorithm without considering the patient's genotype. In contrast, in the PGx-guided dosing group, patients undergo genotype testing, and warfarin is prescribed at a lower dose if they have variant genotypes. Further dose adjustments are based on patients' INR in both groups.

Warfarin therapy is initiated for both groups after MPHV surgery, and their INR is monitored during hospitalization and subsequently through outpatient clinic visits. Patients could either have in-range or out-of-range INR. This is considered in our model to include the probability of adverse events for each state. As shown in Figure 1, we assumed three general health states: Bleeding, thromboembolic events, and no event. The probability of bleeding and thromboembolism is higher in patients with out-of-range INR. Bleeding events include intracerebral hemorrhage (ICH) and extracerebral hemorrhage (ECH). Thromboembolic events include ischemic stroke and transient ischemic attack (TIA). Furthermore, three health states were assumed for those experiencing a stroke: Recovery, sequelae, and death. In comparison, patients suffering from TIA were assumed to recover completely.

### 3.2. Clinical Inputs

Clinical inputs for this study were sourced from published literature. We utilized the primary outcome of the percentage of time in the therapeutic range (PTTR), drawing data from the study by Huang et al. to compare the mean percentage of time spent within or out of the therapeutic range for the two treatment strategies. Patients in the pharmacogenomics (PGx)-guided dosing group experienced a higher PTTR compared to those in the standard dosing group (56.2% vs 44%) (18). The PTTR of INR is a widely applied measure of the quality of vitamin K antagonist anticoagulation management (19), and many prospective clinical trials, often designed to assess the impact of genotype-based dosing on warfarin anticoagulation control, have used PTTR as their primary outcome (20-22).

The risk of thromboembolic and hemorrhagic events was also sourced from the published literature (23). Individuals with in-range INR levels exhibit a lower risk of thromboembolism (2% vs 6%) and bleeding (2.7% vs 9.3%) compared to those with out-of-range INR levels. Of the bleeding events, 29% were classified as ICH, with the remaining 71% being ECH. For those suffering from ICH, we assumed a 48.6% probability of mortality and a 34% probability of recovery with sequelae. Those experiencing ECH had a 5% risk of mortality. Among thromboembolic events, 60% were categorized as stroke, with the remaining 40% being TIA. For patients suffering from stroke, the probabilities of recovery, mortality, and developing sequelae were 44.7%, 11.7%, and 43.6%, respectively. It was assumed that all patients with TIA recovered completely.

This detailed analysis of clinical outcomes based on INR management helps illustrate the potential benefits of PGx-guided dosing in enhancing anticoagulation control and reducing the incidence of adverse events associated with warfarin therapy.

### 3.3. Health State Utilities

The disutility of each health state was sourced from the literature (23). Given the absence of studies on the utility of patients with mechanical heart valves in Iran, we utilized the EuroQol-5D (EQ-5D) questionnaire to measure the baseline utility values for the standard dosing and genotype-guided dosing groups within the Iranian population. After measuring utility weights, we conducted a paired *t*-test to assess the significance of the differences between these utility weights.

A cross-sectional field study was carried out at the Rajaie Cardiovascular Medical and Research Center, located in Tehran, from September to October 2022. This hospital is a leading center for a broad range of cardiovascular diseases, drawing patients from across Iran. The study received ethics approval, and all participating patients provided written informed consent.

The EQ-5D Index, a widely used questionnaire for assessing quality of life (QoL), was employed to calculate utility. This tool records levels of self-reported problems across five dimensions: Mobility, self-care, usual activities, pain/discomfort, and anxiety/depression. We used a Persian-translated version of the EQ-5D Questionnaire to interview patients. The validity and reliability of this translation had been previously tested (24). The responses to the five questions generate 243 possible health states (10), which are then converted into a utility score. In our study, these scores were weighted according to the British value set.

A hypothetical scenario was designed and validated to explain the purpose of the study, outline the challenges, describe possible adverse events of warfarin, and discuss the advantages and disadvantages of pharmacogenomics-guided dosing to the patients.

The investigator, a pharmacist, reviewed participants' medical records for a history of mechanical heart valve replacement before inviting patients to participate in the study. Pregnant individuals, those under 18 years old, and individuals with comorbid diseases were excluded from the study. Before starting the interview, the investigator explained the hypothetical scenario, then read each item of the questionnaire to each participant and recorded their responses.

Due to the lack of reliable data on the prevalence of VHD and the number of mechanical prosthetic heart valve (MPHV) surgeries in Iran, an initial 10 responses to the EQ-5D Questionnaire were recorded. Subsequently, the sample size was determined using Cohen’s f measure and Cochran’s formula, resulting in a required sample of 87 participants with a 95% confidence interval. Ultimately, we collected 105 responses to the EQ-5D Questionnaire.

### 3.4. Costs

All costs were converted from Iranian Rials (IRR) to US Dollars (USD) for the year 2022 using an exchange rate of 279,655 IRR to USD. Due to limitations in calculating indirect medical costs and obtaining accurate estimates of these costs, this study adopts a healthcare sector perspective and considers only direct medical costs (Table 1). Given the incomplete availability of pharmacogenomics tests in Iran, we used the median cost of genotyping tests from other countries as reported in previously published studies. Thus, the median cost of multiple pharmacogenomics tests worldwide was calculated for our model.

**Table 1.** Demographic Data of EQ-5D Questionnaire Respondents

Characteristics	No. (%)
**Sex**	
Male	43 (40.95)
Female	62 (59.05)
**Age (y, mean)**	18 - 72, 50.3 (-)
**Education**	
Primary	54 (51.42)
Senior high	39 (37.14)
Higher education	12 (11.42)
**Marital status**	
Married	73 (69.52)
Unmarried	22 (20.95)
Widow/widower	10 (9.52)
**Smoking status**	
Current smoker	9 (8.57)
Former smoker	14 (13.33)
Never smoker	71 (67.61)
Passive smoker	11 (10.47)
**Comorbidities**	
Atrial fibrillation	8 (7.62)
Hypertension	39 (37.14)
Hyperthyroidism	5 (4.76)
Hypothyroidism	17 (16.19)
Diabetes	21 (20)
HLP	24 (22.85)
Anemia	4 (3.80)
CVA	3 (2.85)
Other	8 (7.6)

Abbreviations: CVA, cerebral vascular accident; HLP: hyperlipidemia.

For each health state defined in our study, we compiled a list of recommended medications, interventions, and services, and cross-checked these with those currently available in Iran to ensure accurate cost measurements. The costs of INR tests, clinical visits for INR monitoring, hospitalization, and management of each health state were obtained from public data provided by Iran's FDA and were based on the approved tariffs for 2022.

### 3.5. Sensitivity Analysis

To determine which parameters our model is sensitive to, a one-way sensitivity analysis was conducted for all model inputs. The ranges for each parameter were obtained from published literature. The incremental cost-effectiveness ratio (ICER) was calculated and compared with the established threshold. If a change in any parameter altered the outcome by more than 20% or affected the ICER significantly, the model was considered sensitive to that parameter.

Additionally, we performed a probabilistic sensitivity analysis to assess the robustness of our model. This analysis helps in understanding the reliability of the model's outcomes under uncertainty, taking into account the variability in key input parameters. This dual approach ensures a comprehensive evaluation of the model's sensitivity to various inputs and its overall stability in providing reliable economic evaluations.

## 4. Results

### 4.1. Utilities

The baseline characteristics of the respondents are presented in Table 1. Results from the EQ-5D Questionnaire indicate that pharmacogenomics (PGx)-guided warfarin dosing increases the utility weight from 0.696 ± 0.238 to 0.760 ± 0.236, with a confidence interval of 95%. Additionally, the results of a paired *t*-test indicate that the difference between the utility weights is statistically significant (P-value < 0.0001). The responses to the EQ-5D Questionnaire for both the standard dosing and pharmacogenomics-guided dosing arms are detailed in Appendix 1.

### 4.2. Base-Case Analysis

Table 2 presents the utilities and disutilities that were incorporated into the model. Our analysis revealed that, compared to standard warfarin dosing, genotype-guided dosing increases quality-adjusted life years (QALYs) by 0.07 and costs by approximately $177. The ICER was calculated to be $2474 per QALY gained, which exceeds the pharmacoeconomic threshold for medical intervention in Iran's healthcare system, set at $1500.

**Table 2.** Input Parameters of Decision Tree Model

Parameter	Probability	95% Confidence Interval (CI)	References
**Probabilities**			
INR in range (in standard dosing group)	0.44	0.242 - 0.634	(18)
INR in range (in genotype - guided dosing group)	0.562	0.37 - 0.754	(18)
INR out of range (in standard dosing group)	0.56	calculated	(18)
INR out of range (in genotype - guided dosing group)	0.438	calculated	(18)
Thromboembolism (within therapeutic INR range)	0.02	0.01 - 0.03	(23)
Thromboembolism (out of therapeutic INR range)	0.06	0.03 - 0.1	(23)
Bleeding (within therapeutic INR range)	0.027	0.013 - 4.02	(23)
Bleeding (out of therapeutic INR range)	0.093	0.022 - 0.093	(23)
ICH	0.29	0.23 - 0.35	(25)
ECH	0.71	-	(25)
Stroke	0.6	0.48 - 0.72	(25)
TIA	0.4	-	(25)
**Utilities**			
Standard dosing	0/696	0.458 - 0.934	Calculated
Genotype - guided dosing	0/760	0.524 - 0.996	Calculated
ICH	- 0.14	- 0.12 to - 0.16	(25)
ECH	- 0.06	- 0.02 to - 0.1	(25)
TIA	- 0.103	- 0.088 to - 0.119	(25)
Stroke	- 0.14	- 0.12 to - 0.16	(25)
Sequelae	- 0.374	- 0.16 to - 0.374	(25)
Death	0.0	-	(25)
**Costs**			
Genotyping	187.5	-	National tariffs
INR monitoring	16	-	National tariffs
ICH	241	-	National tariffs
ECH	205	-	National tariffs
TIA	178	-	National tariffs
Stroke	311	-	National tariffs
Sequelae	21.5	-	National tariffs

Abbreviations: ECH, extracranial haemorrhage; ICH, intracranial haemorrhage; INR, international normalized ratio; TIA transient ischemic attack.

### 4.3. One-way Sensitivity Analysis

**Figure 2.**
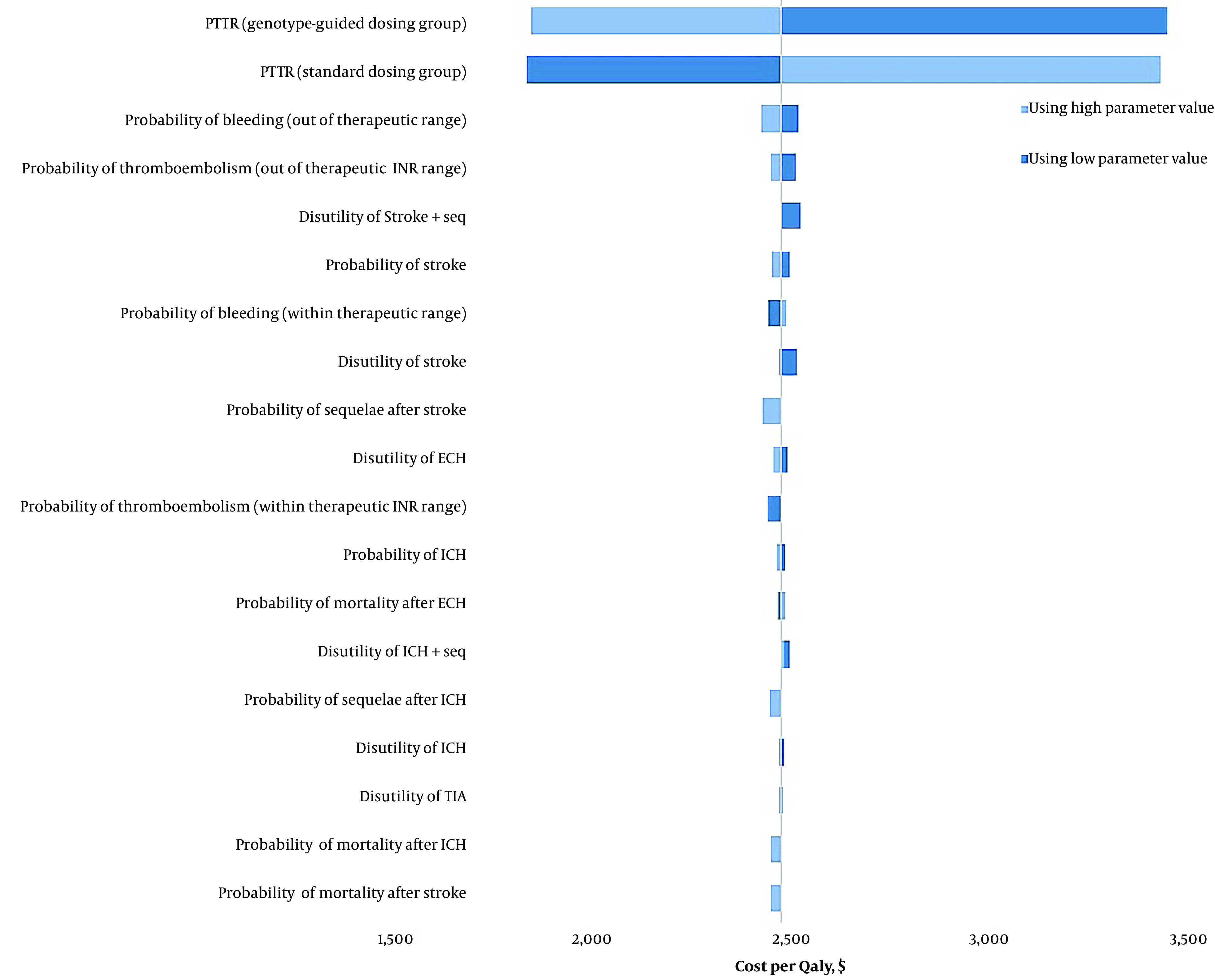
Tornado diagram for one-way sensitivity analysis. ECH, extracranial haemorrhage; ICH, intracranial haemorrhage; INR, international normalized ratio; PTTR, percentage of time in therapeutic range; QALY, quality-adjusted life-year; seq, sequelae; TIA, transient ischemic attack.

We conducted a one-way sensitivity analysis on the model's parameters, and the results are depicted in Figure 2 through a tornado diagram. The model demonstrated no sensitivity to the disutilities and probabilities of adverse events. However, the PTTR for both treatment groups significantly influenced the cost-effectiveness, increasing the ICER by more than 20%.

Specifically, when the PTTR for the standard dosing group was varied from 0.24 to 0.63, the ICER ranged from $1833 to $3424 per QALY gained. Conversely, when the PTTR for the PGx-guided dosing group was adjusted from 0.3 to 0.7, the ICER ranged from $3441 to $1843 per QALY gained. Additionally, one-way sensitivity analyses were conducted on the costs associated with ICH, ECH, stroke, TIA, and sequelae. Although these cost adjustments increased the cost per QALY, they did not significantly impact the robustness of our model.

The sensitivity analysis of the utility weights revealed that the model was sensitive to the utility of both groups. When the utility weight of the standard group was set to its lower limit, the ICER decreased to $602 per QALY, making it cost-effective. Conversely, setting it to its upper limit resulted in an ICER of -$1174. For the PGx-guided group, setting the utility weight to its lower limit resulted in an ICER of -$1166, and setting it to its upper limit brought the ICER to $600, which was also cost-effective.

Different utility values were calculated for the standard dosing and pharmacogenomics-guided dosing arms, while other cost-effectiveness studies have used the same utility weights in their models. A sensitivity analysis using the utility weights from the Kim et al. study (25) calculated an ICER significantly higher than Iran's pharmacoeconomic threshold ($18396), indicating it was not cost-effective.

The cost of the PGx test also influenced the model's sensitivity; if the cost of the PGx test decreased to $118, the ICER would drop below Iran's pharmacoeconomic threshold and become cost-effective.

### 4.4. Probabilistic Sensitivity Analysis

**Figure 3.**
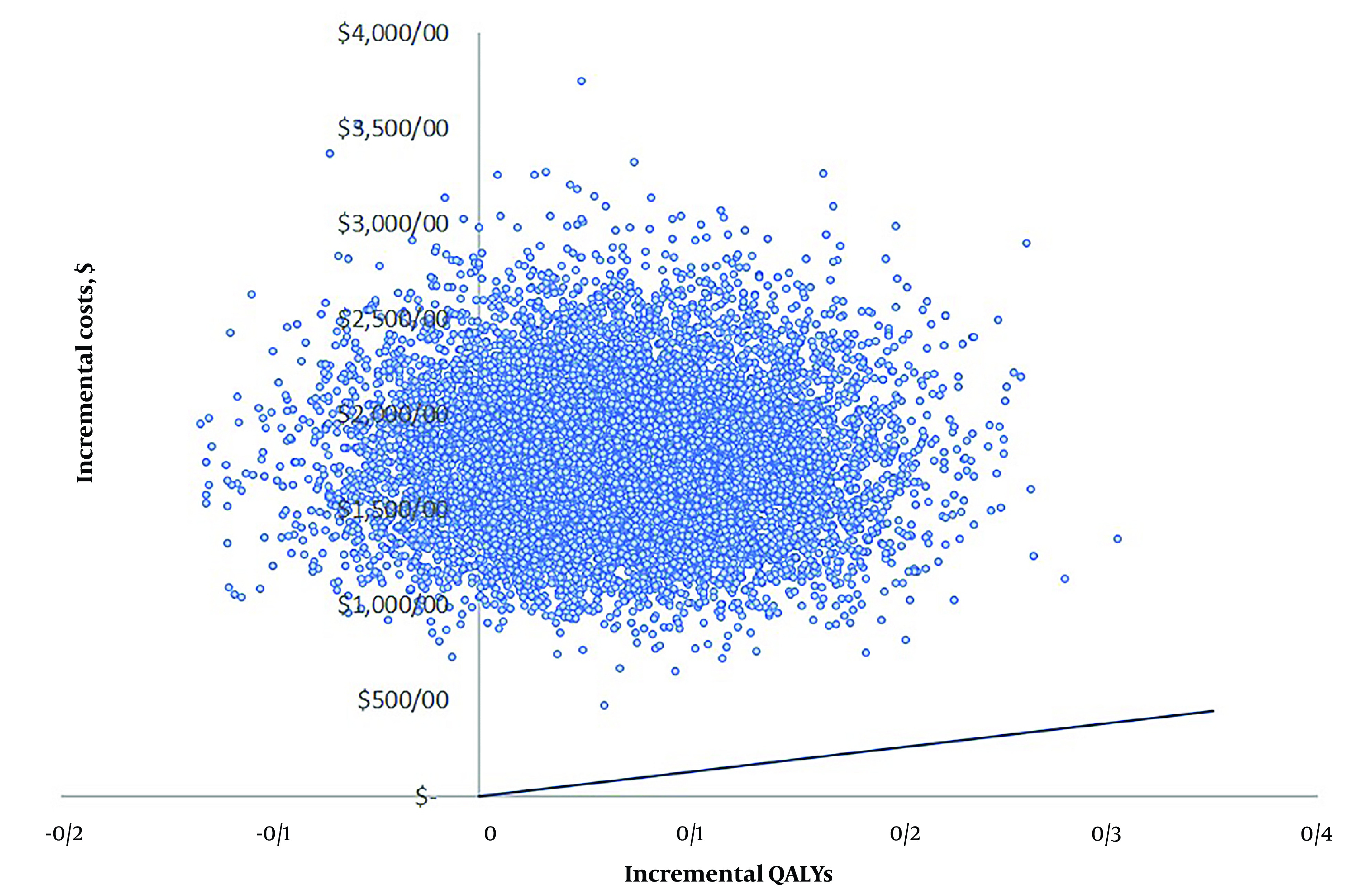
Probabilistic sensitivity analysis. QALY, quality-adjusted life-year.

To evaluate the effect of uncertainty of all model inputs, we performed a probabilistic sensitivity analysis using Monte Carlo simulation. Figure 3 illustrates the distribution of the ICER from 10,000 model runs conducted in Excel 2022. The majority of these points (86.1%) fall into the upper right-hand quadrant of the cost-effectiveness plane, indicating that dosing of warfarin using genotyping is more costly than standard care and are above Iran’s pharmacoeconomic threshold.

## 5. Discussion

Our analysis indicates that pharmacogenetic-guided warfarin dosing improves patients' QoL but results in a higher cost. The calculated ICER was $1500 per QALY, which exceeds Iran’s pharmacoeconomic threshold, showing pharmacogenetic-guided dosing is not cost-effective. Despite 86.1% of the probabilistic sensitivity analysis simulations showing that PGx-guided dosing is not cost-effective and confirming the robustness of the model, the deterministic sensitivity analysis revealed that the model is sensitive to the PTTR, the cost of the pharmacogenetic test, and the utility values of both standard dosing and pharmacogenetic-guided dosing groups.

Regarding PTTR, altering this parameter did not reduce the ICER to below Iran’s pharmacoeconomic threshold but did result in a change of more than 20% in the ICER, highlighting the model's sensitivity to this input. As for the cost of the pharmacogenomics test, if it decreases to less than $118, pharmacogenomics-guided dosing would become cost-effective. Additionally, if the utility of the pharmacogenomics-guided dosing group increases to 0.86, this strategy will also become cost-effective.

Previous cost-effectiveness analyses of pharmacogenomics-guided dosing of warfarin have produced mixed results. A systematic review conducted by You (26) on economic evaluation studies concerning pharmacogenomics tests for individuals requiring anticoagulant therapy reported that the ICER in all four studies exceeded $50,000 USD, indicating that such interventions were not cost-effective. Likewise, other later published studies have calculated ICERs that were not cost-effective (23, 27). However, a study conducted in England reported that using genetic tests for warfarin dosing was cost-effective, with an ICER of 13,226 pounds per QALY gained (28). Similarly, a study by Kim et al. (25), which targeted the same patient group as our study, found that pharmacogenomics-guided dosing is cost-effective despite the minor frequencies of sensitive alleles in CYP2C9 and VKORC1.

We opted for a 1-year time horizon for our model, in line with previous studies (29, 30), primarily because the highest incidence of thrombosis and related side effects occurs during the first year post-surgery (31, 32). Clinicians noted that patients struggle most with warfarin's side effects during this period due to unfamiliarity with managing warfarin therapy and monitoring INR. Recovery from surgery also makes patients more prone to side effects within the first year. Genetic specialists emphasized that genetic testing is most effective if conducted early before starting warfarin, as it helps identify sensitive patients promptly. Delaying testing diminishes its impact, as sensitive patients are likely identified within six months post-surgery through INR monitoring or from experiencing side effects like bleeding.

The study found pharmacogenomics-guided dosing of warfarin is not cost-effective mainly because the costs associated with warfarin's adverse events are lower in Iran compared to other countries, and while only a proportion of patients experience these events, genetic testing would be administered to all patients at a high cost. However, with a downward trend in the prices of pharmacogenomics tests (33, 34), using pharmacogenomics-guided warfarin dosing could become cost-effective in the upcoming years in Iran.

The significance of pharmacogenomics in medical treatment extends beyond just clinical outcomes and improved utility; it encompasses additional values that may not be directly reflected in increased utility scores. For instance, the concept of process utility, which is the value derived from the method used to achieve a health outcome, plays a crucial role in decision-making regarding medical interventions (35, 36). In the context of pharmacogenomics-guided dosing of warfarin, when patients were informed about the benefits of this approach, such as a reduced chance of bleeding and thromboembolic events and quicker achievement of therapeutic INR levels compared to standard dosing, they reported a reduction in anxiety regarding treatment. This response highlights how personalized treatment can enhance patient comfort and confidence, even though they had not experienced this intervention firsthand but only understood its potential benefits. By presenting a hypothetical scenario before administering the questionnaire, we were able to incorporate process utility into our study, leading to higher utility scores for the pharmacogenomics-guided dosing arm.

Additionally, Goring et al. (37) presented the "value flower," which introduces 12 elements to be considered in cost-effectiveness analyses, including "the value of reduction in uncertainty." This concept emphasizes the importance of diagnostic tests that help predict treatment responses, thereby potentially avoiding costs associated with adverse drug reactions. In the case of warfarin therapy, genotype testing can identify normal and poor metabolizers, allowing physicians to tailor monitoring frequency and reduce the risk of adverse events for certain patients. Our study factored in the fewer warfarin-associated adverse events among poor metabolizers, which resulted in lower overall costs for adverse events in the pharmacogenomics-guided dosing arm compared to the standard dosing arm. This integration of pharmacogenomics thus not only improves treatment efficacy but also enhances economic evaluations by reducing uncertainty and personalizing patient care.

Utilizing pharmacogenomics can enhance the certainty of treatment efficacy for normal metabolizers, potentially increasing their adherence to prescribed treatments—an additional value component as per Goring et al. (37). Genotyping can thus be seen as a factor that improves adherence, which in turn may prevent the costs associated with poor adherence. Incorporating this effect into cost-effectiveness analyses could significantly alter the outcomes of economic models.

Traditionally, research topics and processes were defined by researchers and policymakers. However, there has been a shift towards incorporating patients' perspectives into research to ensure that their needs and challenges are comprehensively addressed (38-41). In recognition of the benefits of patient engagement, our study took a proactive approach by directly interviewing patients and assessing their QoL. We propose that, rather than uniformly applying pharmacogenomics tests across the board (or eschewing them entirely, depending on each country's healthcare policy), it may be more effective to involve patients in the decision-making process regarding their treatment. This could involve informing them about the potential advantages and disadvantages of pharmacogenomics tests, consulting with their physician, and allowing them to decide whether to proceed with testing.

Given the potential benefits that pharmacogenomics can bring to clinical practice, its economic impact is garnering attention globally. However, to our knowledge, there are only a few cost-effectiveness studies that have evaluated the economic implications of pharmacogenomics-guided treatments in Iran, such as the study on genotype-guided fluoropyrimidine-based chemotherapy (42). This study represents the first cost-effectiveness analysis of pharmacogenomics in the field of cardiovascular diseases in Iran. We recommend further research to explore the clinical and economic effects of pharmacogenomics on the management of various diseases, which could aid Iranian policymakers in making informed decisions about the implementation of pharmacogenomics in clinical practice.

This study has several notable strengths. We utilized a validated EQ-5D Questionnaire and a hypothetical scenario in Persian to directly interview Iranian patients with MPHV. This approach ensured that the calculated utilities are specifically tailored for our target demographic—Iranian patients with MPHV—enhancing the accuracy of our model's results. We exclusively included patients who had recently undergone mechanical heart valve replacement surgery and excluded those who had the surgery more than a year ago. This is based on evidence suggesting that patients are most sensitive to complications during the first year post-surgery, a period when genetic testing can be particularly crucial.

However, one significant limitation of our study is the absence of localized data on the impact of pharmacogenomics-guided dosing of warfarin on the PTTR and the probability of adverse events for the Iranian population. Consequently, we relied on data from published literature pertaining to other populations. This reliance on non-local data may affect the applicability and precision of the findings specifically to the Iranian context, underscoring the need for more region-specific research in this area.

ijpr-23-1-143898-s001.pdf

## Data Availability

The data presented in this study are uploaded during submission as a supplementary file and are openly available for readers upon request.
