# Supplementary material for: Cost-Effectiveness Analysis of Pharmacogenomics-Guided Versus Standard Dosing of Warfarin in Patients with Mechanical Prosthetic Heart Valve
Source: Iran J Pharm Res. 2024 May 14;23(1):e143898. doi: 10.5812/ijpr-143898 (PMC11302438; doi:10.5812/ijpr-143898)
Supplement: ijpr-23-1-143898-s001.pdf [file ijpr-23-1-143898-s001.pdf]

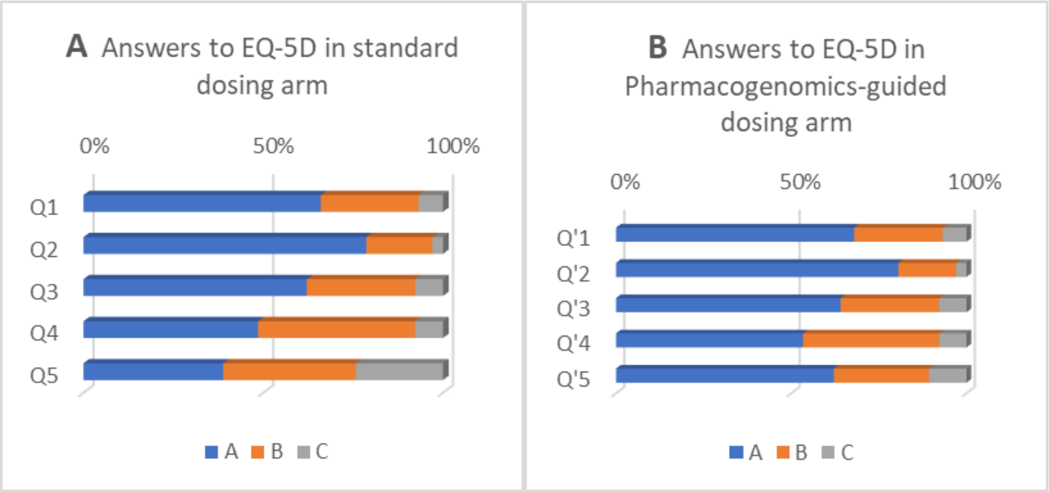


**Appendix 1. Respondents' answer to each question of the EQ-5D questionnaire for standard dosing arm (A) and pharmacogenomics-guided dosing arm (B).** The EQ-5D questionnaire contains 5 questions. Each question measures a dimension of health in 3 levels of severity. In Figure 1, questions 1-5 refer to mobility, self-care, usual activities, pain/discomfort and anxiety/depression, respectively. Choices A, B and C represent no, moderate, or extreme problems in each dimension. Figure 1 demonstrates that pharmacogenomics-guided dosing has a higher beneficial effect on anxiety or depression (question 5) as the number of patients that do not feel anxious/depressed increases by 22.85% after taking genotyping tests. In comparison, pharmacogenomics-guided dosing does not have a considerable effect on other dimensions of patients' quality of life.

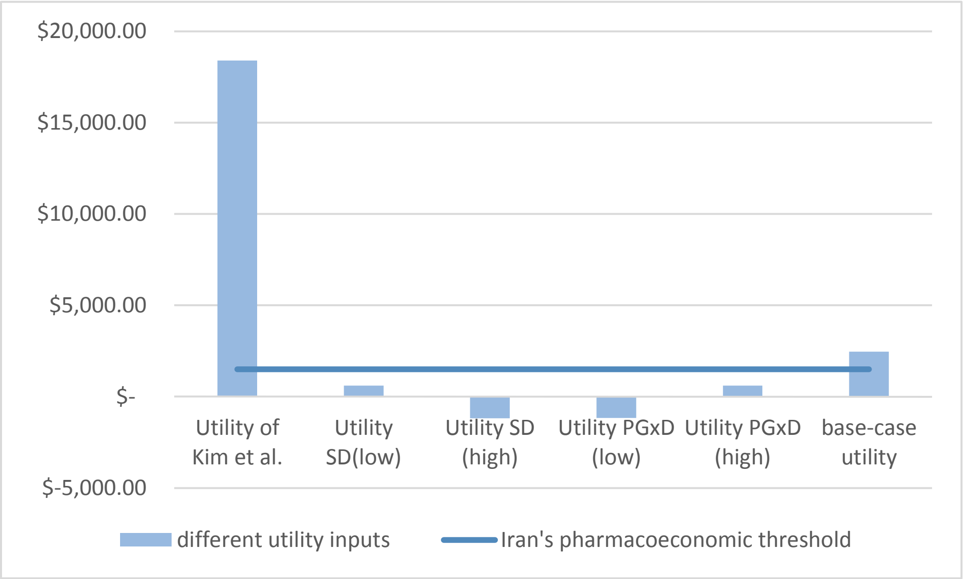

**Appendix 2. Bar chart of sensitivity analysis for utility.**

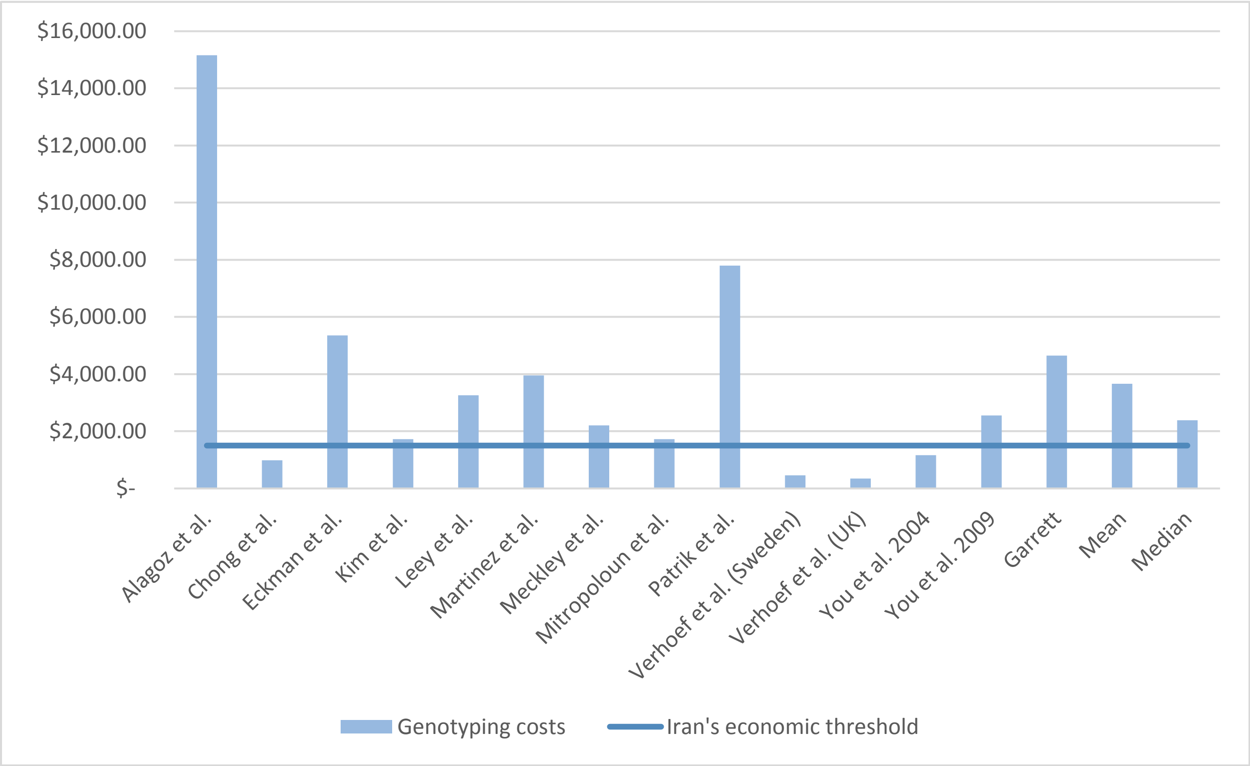


**Appendix 3. Bar chart of sensitivity analysis for genotyping test cost.**
